# Comparison of practice based research network based quality improvement technical assistance and evaluation to other ongoing quality improvement efforts for changes in agency culture

**DOI:** 10.1186/s12913-015-0956-3

**Published:** 2015-07-31

**Authors:** William C. Livingood, Angela H. Peden, Gulzar H. Shah, Nandi A. Marshall, Ketty M. Gonzalez, Russell B. Toal, Dayna S. Alexander, Alesha R. Wright, Lynn D. Woodhouse

**Affiliations:** Center for Health Equity & Quality Research, University of Florida College of Medicine-Jacksonville, 580 West 8th St, Tower II, Suite 6015, Jacksonville, FL 32082 USA; Jiann Ping Hsu College of Public Health, Georgia Southern University, Statesboro, GA USA; Department of Health Sciences, Armstrong State University, Savannah, GA USA; East Central Health District (retired), District 6, Augusta, GA USA; Division of Pharmaceutical Outcomes & Policy, UNC Eshelman School of Pharmacy, Asheville, NC USA

**Keywords:** Quality improvement, Organizational culture, Practice based research network, Public health agency, Local health department

## Abstract

**Background:**

Public health agencies in the USA are increasingly challenged to adopt Quality Improvement (QI) strategies to enhance performance. Many of the functional and structural barriers to effective use of QI can be found in the organizational culture of public health agencies. The purpose of this study was to assess the impact of public health practice based research network (PBRN) evaluation and technical assistance for QI interventions on the organizational culture of public health agencies in Georgia, USA.

**Methods:**

An online survey of key informants in Georgia’s districts and county health departments was used to compare perceptions of characteristics of organizational QI culture between PBRN supported QI districts and non-PBRN supported districts before and after the QI interventions. The primary outcomes of concern were number and percentage of reported increases in characteristics of QI culture as measured by key informant responses to items assessing organizational QI practices from a validated instrument on QI Collaboratives. Survey results were analyzed using Multi-level Mixed Effects Logistic Model, which accounts for clustering/nesting.

**Results:**

Increases in QI organizational culture were consistent for all 10- items on a QI organizational culture survey related to: leadership support, use of data, on-going QI, and team collaboration. Statistically significant odds ratios were calculated for differences in increased QI organizational culture between PBRN-QI supported districts compared to Non-PBRN supported districts for 5 of the 10 items, after adjusting for District clustering of county health departments.

**Conclusions:**

Agency culture, considered by many QI experts as the main goal of QI, is different than use of specific QI methods, such as Plan-Do-Study-Act (PDSA) cycles or root-cause analyses. The specific use of a QI method does not necessarily reflect culture change. Attempts to measure QI culture are newly emerging. This study documented significant improvements in characteristics of organizational culture and demonstrated the potential of PBRNs to support agency QI activities.

## Background

Quality Improvement (QI) is part of the USA’s strategy for improving local public health systems [1, 2]. These QI strategies for public health are frequently integrated with the “essential services” that serve as a foundation for both measurement of agency performance [3, 4] and agency accreditation [5]. Some challenges to adopting QI are ***structural*** such as inadequate economies of scale supporting public health essential services [6]. QI work with Florida and Georgia local public health systems have also revealed other common, more ***functional*** challenges to public health agency use of QI principles [7–9], including: traditional administration and management practices based on hierarchical decision making, emphasis on rigidly following rules rather than outcomes, narrowly defined and siloed (not my job) work responsibilities, and “once and done” approach to problems rather than continuous improvement [10, 11]. Many of these functional barriers reflect what might be considered the organizational culture of agencies, the shared values, beliefs, and norms of the agency which are commonly defined characteristics of organizational culture [12–14].

Despite major structural and functional challenges, local agencies pursuing accreditation are being required by the Public Health Accreditation Board (PHAB) to use QI methods and techniques as a part of a QI Plan. QI is also embodied in the most recent standards and measures of local public health accreditation [15]. PHAB’s requirements followed the growing importance of QI for improving the quality of health care [5] and the need for public health to adopt QI to improve performance and effectiveness [16]. However, public health’s success in adopting QI is not well established [17], and with few exceptions, the impact on changing organizational culture to reflect QI principles and practices is even less reported.

A primary purpose of this study was to assess the impact of public health practice based research network (PBRN) QI interventions on the organizational culture of selected Georgia public health districts. The perceived culture changes of districts receiving the QI interventions (PBRN-QI) were compared to districts not receiving the QI interventions (Non-PBRN) using a structured tool for measuring organizational culture based on QI principles.

## Methods

### Design

The primary research question focused on assessing culture change associated with QI study/intervention by the Georgia Public Health Practice-Based Research Network (PBRN), within selected health districts in GA, comparing perceptions of QI organizational cultural characteristics of the three PBRN-QI districts to 10 Non-PBRN districts (comparison group). Consequently, perceptions by key informants of local agency functioning that reflect QI culture were the focus of this survey research. The primary data collection tool was a web-based survey instrument, designed to assess the level of improvement in organizational QI culture over a 12 month period in the PBRN-QI districts, compared with change in QI culture in the Non-PBRN agencies during the same time period. The ten-item survey came from an instrument that was validated and adapted for studying districts as quality improvement collaboratives (QIC) in Georgia [18, 19]. Psychometrics for the original instrument [18] primarily focused on QI content (using expert panels) and construct validity (using exploratory factor analysis) for clinical settings in addition to testing for reliability (using Cronbach’s alpha analysis). The revised instrument adapted for public health collaboratives in Georgia was primarily tested for validity and reliability for public health content using an expert panel and Cronbach’s alpha analysis [19]. The content of the ten specific QI organizational culture items was previously identified in an evaluation of QI interventions in a Florida county health department [10]. This adapted survey was administered to key informants in each district to obtain their perceptions of the current and prior year’s status of organizational characteristics of QI culture. The same respondents completed the pre and post assessments.

### Setting/participants

Georgia’s local public health system is based statutorily on county health departments (CHDs). However, 18 public health districts have emerged as the primary mechanism for organizing CHD services across the 159 counties in Georgia [19]. All 18 districts were invited to participate in the PBRN, and 13 of the 18 districts were actively engaged in the initial PBRN survey of public health agency QI, including 118 CHDs [19]. In effect, the district participation rate for these QI studies was 72 %. Three districts (3 of the 6 founding district members of the PBRN) volunteered to participate in the PBRN sponsored QI intervention (QI technical assistance and evaluation study), covering 37 counties in the southeastern part of the state. Fifteen counties in the three QI supported districts were in the bottom quartile of Georgia’s County Health Rankings and 5 were in the upper quartile [20]. The ten other PBRN participating districts were invited and agreed to participate in the survey of QI culture, thereby serving as the comparison group. (See Table 1 for PBRN QI supported districts versus comparison districts for number of counties in districts, population ranges of counties in each district, and range of health rankings for counties in each district). The key informants in each district, including both county and district personnel, were previously identified by the district leadership (district director or their designee) [11, 19] or were identified by PBRN staff as QI leadership within the districts. Invitations to participate in this survey were sent to 76 key informants from the QI intervention districts and 120 key informants from the non-QI intervention districts (10 districts covering 83 counties).

**Table 1:** Characteristics of participating districts

Participating districts	# of counties in each district	Population range of counties in each district	Range of county health rankings
PBRN Supported Districts			
East Central 6	13	1680–131,627	7–158
Coastal 9-1	8	13,839–276,434	13–73
Southeast 9-2	16	6718–72,694	53–154
Non-PBRN Supported Districts			
North Georgia 1-2	6	23,760–225,106	6–114
North 2	13	10,771–195,405	1–118
Fulton 3-2	1	977,773	20
Clayton 3-3	1	265,888	38
DeKalb 3-5	1	713,340	19–126
South Central 5-1	10	8913–48,041	54–140
North Central 5-2	13	8447–156,462	12–142
West Central 7	16	2404–198,413	8–156
South 8-1	10	3988–114,552	50–150
Southwest 8-2	14	3366–94,501	37–151

### Intervention

PBRNs originated with clinical practices where they have demonstrated over decades that that they can make major and unique contributions in improving clinical practice [21–25]. PBRNs are a recently emerging approach in public health settings with growing utility [26–29]. Increased research related capacity of PBRNs to enhance services [30] and improve data and surveillance [31] has also been demonstrated with clinical practice PBRNs. The use of PBRNs to support quality improvement in local public health agencies is an example of enhanced research related capacity to improve services, albeit not research in itself.

For these QI interventions, the PBRN worked with each of the selected QI intervention districts to select a focus for a QI initiative. This process of identifying the focus of QI required distinguishing between improving an on-going service versus a one-time initiative that would not be appropriate for QI. The *Model for Improvement* [32] was selected as the QI approach for the interventions, which is primarily characterized as the use of Plan-Do-Study-Act (PDSA) cycles for QI. QI techniques included the use of Root Cause Analysis [33], process mapping [34], and variations of data displays and control charts [35].

These QI initiatives were supported by a RWJF grant to support development of aspiring Public Health Systems and Services Research (PHSSR) researchers. Development of QI research/evaluation capacity involved training and education of these PHSSR researchers in public health QI technical assistance. Three aspiring DrPH students were selected and assigned to the State Coordinating Center for GA Public Health PBRN [36] to provide technical assistance and evaluation for the three districts. Their training and education included 1) weekly seminar meetings using QI textbooks, reports in journals, and other examples of QI applications in public health; 2) Collaboration with the University of Minnesota Public Health QI Center to build capacity, and 3) a six sigma certification process [37] through online training [11]. In addition to education and training provided by the PBRN coordinating center, the Doctoral students provided on-site QI technical assistance and liaison with the PBRN State Coordinating Center in addition to conducting on site observations for evaluation and feedback to the coordinating Center.

The three participating health districts selected county health department level QI projects. District A focused on increasing HIV performance measures in two county wellness centers. Using PDSA, root cause analysis and process mapping, inaccurate data entry was determined to be the cause of this problem, resulting in corrective action that achieved major improvements in data entry [38]. District B focused on decreasing teen clinic wait times in a local health department. Although this QI project did not achieve the goals for decreased wait times during the study period, participants reported learning QI techniques and the need to overcome challenges within their organization [39]. They also reported plans to conduct additional QI projects. District C focused on increasing HIV testing in a local health department. An increase in testing was observed but this project struggled with overcoming the challenge of stopping work on the project at the end of a planned time period rather than continuous work until optimal results were achieved.

The approach to combining technical assistance with evaluation described above reflects a developmental evaluation design, wherein the evaluators use formative and summative information from the evaluation sites to provide feedback and input to help maximize the results [40–42]. This evaluation approach is more appropriate for QI related evaluation, applied research, practice based research designs, and implementation science designs where maximizing results and optimal improvement are as important as answering the research question. It is also consistent with a primary mission of PBRNs to improve practice.

### Data collection

The primary outcomes (changes in perceived QI culture) were measured through a survey instrument using selected items from the QIC assessment instrument developed by Schouten [18] and validated for public health agencies in Georgia [19]. Using ten selected items focused on characteristics of organizational QI culture [10], data were collected through Qualtrics, an online survey tool (http://www.qualtrics.com). Data were collected and analyzed in 2013. The principles of QI that the questions address and the cultural characteristics that are challenges to a QI culture are displayed in Table 2, including each survey item number. The specific items are shown in Table 2 in the Results section.

**Table 2:** Relationship of QI principles, barriers and QI culture survey items

QI organizational principles	QI organizational antithesis	Items
Leadership supports QI principles and practices	Administration and management emphasize hierarchical decision making and administrative procedures	1, 2, 3
Use of data to inform decision making and employee behavior	Emphasis on rigidly following rules rather than producing outcomes	5, 6
Staff regularly engage in team problem solving and collective efforts to achieve organizational outcomes	Narrowly defined and siloed (not my job) work responsibilities.	4, 10
Ongoing (continuous) monitoring and performance	Once and done	7, 8, 9

This research was reviewed and approved by the Georgia Southern University Human Subjects Institutional Review Board (IRB).

#### Analysis

Changes in key informant perceptions of QI culture were analyzed using a three step process:

Step 1: Differences were computed for each of the ten items by subtracting perceptions of QI characteristics a year ago from perceptions of current QI characteristics.

Step 2: Responses were re-coded for the above change in perception score over 12 months into a dichotomous variable. Public health districts with positive change (e.g. score of “agree” 12 months ago changed to “strongly agreed” in the current measure) were coded 1 to indicate “improvement during 12 months”, those with no change or negative change were coded as 0 to indicate “No improvement during 12 months”.

Step 3: Odds ratios to show “improvement during 12 months” were computed for the key informant responses for the CHDs and districts that participated in the PBRN’s QI projects compared to the Non-PBRN key informant responses. The Odds Ratio was selected as the statistical test to document “effect size” in contrast to the “t” test or ANCOVA because of: greater detail on effect size beyond significance (*p* value), different starting points, and the dichotomous dependent variable (improvements in characteristics of QI culture) [43, 44]. Since the respondents for this study came from 13 health districts in Georgia, we anticipated that clustering would impact the effect size with substantial potential to reduce statistical significance. To assess the impact of clustering and to deal with multilevel data, we performed the multi-level mixed effects logistic model (xtmelogit) using Stata/SE 11.2 for Windows (2009 StataCorp LP) with Adaptive Gauss-Hermite quadrature approximation (to improve the accuracy of estimation). Since the multilevel modeling showed clustering effect in most of the items, the final odds ratios reported in the Table 2 are from the multi-level model, rather than those from single level logistic function. *T*-test was used to calculate the proportion of health districts that had improved on each dimension of QI for descriptive statistical purposes.

## Results

A total of 147 of the key informants responded, representing all 13 of the 18 districts participating in the initial survey of Georgia public health districts related to districts functioning as QICs and providing public health essential services [19]. Of 120 invited key informants, from the Non-PBRN districts, 85 responded to the online survey, whereas 62 of 76 invited key informants responded from the PBRN-QI districts. The overall participation rate of invited key informants was 75 %.

This comparison of perceptions of QI culture showed that the odds of improved QI cultural characteristics were significantly greater (OR ranging from 1.97 to 10.14) for health districts that participated in the Georgia PBRN-QI activities, reflecting a .05 level or better significant increase in support for five of the 10 QI domains and a .10 level of significant increase for eight of the 10 domains. These domains of QI are reflected in the 10 items in Table 3. The time frame for the change in level of agreement was **12 months prior** to the survey of health districts, and the change was operationalized by asking the level of agreement to the statements about QI organizational characteristics 12 months prior to the survey versus the level of agreement with **current** QI characteristics.

**Table 3:** Comparison of change/increase^a^ in self-assessed organizational characteristics of QI culture and odds ratios^b^ for whether HEALTH DISTRICTs improved in quality improvement characteristics during 12 months

Quality improvement characteristic	PBRN QI change^a^	NO PBRN QI change^a^	Odds ratios	*P* value
1. “My Health District supports the goals of public health essential services quality improvement”	47 %	15 %	10.135	0.000
2. “Health District management prioritizes success for public health essential services quality improvement”	40 %	6 %	2.684	0.095
3. “Health District staff are motivated in implementing changes for quality improvement”	49 %	10 %	4.954	0.006
4. “Participation in public heath essential services quality improvement enhances multidisciplinary collaboration in my organization”	58 %	0 %	5.147	0.007
5. “Public heath essential services quality improvement goals are readily measurable”	42 %	3 %	3.824	0.024
6. “Our Health District staff work with County Health Department staff to use measurements to plan change”	47 %	13 %	2.959	0.058
7. “Our Health District staff, working with the County Health Department staff, considers continuous QI part of the public health agency’s working process.”	53 %	16 %	2.875	0.060
8. “Our Health District staff, working with the County Health Department staff, continues to aim for change”	48 %	13 %	1.968	0.218
9. “Our Health District staff work with the County Health Department staff to track progress continuously”	34 %	4 %	4.710	0.011
10. “Information, ideas, and suggestions are actively exchanged at quality improvement meetings”	48 %	15 %	2.122	0.176

^a^Percent change form undecided, disagree, or strongly disagree to agree or strongly agree

^b^Odds ratio Improvements calculated from each increase in self assessed 5 point ordinal score

### Leadership support for QI

Significantly more key informants from health districts that participated in the PBRN sponsored QI (PBRN-QI) initiative reported that their health district’s level of support increased for quality improvement in public health essential services in the past 12 months. The odds for this increase in this QI (Item 1) characteristic (47 % increase) were 10.14 times greater (*p* = .000) for the health districts participating in the PBRN’s QI initiative than health districts that did not participate in the PBRN QI (Non-PBRN) initiative (15 % increase). See Table 4 for the Comparison of change between the two groups with the odds ratios and *p* values.

**Table 4:** Responses to each item of QI culture by QI PBRN district key informants versus comparison district key informants

Quality improvement characteristic		Highly disagree		Disagree		Undecided		Agree		Highly agree		*Mean*	
		1 year ago	Currently	1 year ago	Currently	1 year ago	Currently	1 year ago	Currently	1 year ago	Currently	1 year ago	Currently
1. “My Health District supports the goals of public health essential services quality improvement”	Intervention	5 (8.3 %)	4 (6.5 %)	4 (6.7 %)	2 (3.2 %)	9 (15 %)	2 (3.2 %)	22 (36.7 %)	22 (35.5 %)	20 (33.3 %)	32 (51.6 %)	3.80	4.23
	Control	4 (4.9 %)	7 (6 %)	7 (6.2 %)	6 (7.4 %)	12 (14.8 %)	8 (6.9 %)	26 (32.1 %)	37 (31.9 %)	33 (40.7 %)	57 (49.1 %)	3.96	4.12
2. “Health District management prioritizes success for public health essential services quality improvement”	Intervention	5 (8.3 %)	5 (8.1 %)	6 (10 %)	3 (4.8 %)	14 (23.3 %)	7 (11.3 %)	20 (33.3 %)	23 (37.1 %)	15 (25 %)	24 (38.7 %)	3.57	3.94
	Control	4 (4.9 %)	5 (5.9 %)	6 (7.3 %)	6 (7.1 %)	14 (17.1 %)	11 (12.9 %)	31 (37.8 %)	32 (37.6 %)	31 (36.5 %)	42 (35.9 %)	3.87	3.92
3. “Health District staff are motivated in implementing changes for quality improvement”	Intervention	7 (11.9 %)	5 (8.2 %)	4 (6.8 %)	2 (3.3 %)	15 (25.4 %)	8 (13.1 %)	19 (32.2 %)	22 (36.1 %)	14 (23.7 %)	24 (39.3 %)	3.49	3.95
	Control	2 (2.5 %)	5 (6.1 %)	10 (12.5 %)	5 (6.1 %)	16 (20 %)	15 (18.3 %)	30 (37.5 %)	29 (35.4 %)	22 (27.5 %)	28 (34.1 %)	3.75	3.85
4. “Participation in public heath essential services quality improvement enhances multidisciplinary collaboration in my organization”	Intervention	6 (10 %)	3 (4.8 %)	5 (8.3 %)	3 (4.8 %)	7 (11.7 %)	4 (6.5 %)	28 (46.7 %)	21 (33.9 %)	14 (23.3 %)	31 (50 %)	3.65	4.19
	Control	3 (3.7 %)	5 (5.9 %)	11 (13.6 %)	10 (11.8 %)	14 (17.3 %)	14 (16.5 %)	35 (43.2 %)	34 (40 %)	18 (22.2 %)	22 (25.9 %)	3.67	3.68
5. “Public heath essential services quality improvement goals are readily measurable”	Intervention	4 (6.7 %)	3 (4.8 %)	7 (11.7 %)	5 (8.1 %)	21 (35 %)	9 (14.5 %)	18 (30 %)	31 (50 %)	10 (16.7 %)	14 (22.6 %)	3.38	3.77
	Control	2 (2.5 %)	3 (3.6 %)	10 (12.5 %)	11 (13.3 %)	29 (36.3 %)	23 (27.7 %)	30 (37.5 %)	36 (43.4 %)	9 (11.3 %)	10 (12 %)	3.43	3.47
6. “Our Health District staff work with County Health Department staff to use measurements to plan change”	Intervention	6 (10 %)	4 (6.5 %)	8 (13.3 %)	4 (6.5 %)	17 (28.3 %)	10 (16.1 %)	19 (31.7 %)	29 (46.8 %)	10 (16.7 %)	15 (24.2 %)	3.32	3.76
	Control	3 (3.8 %)	5 (6.1 %)	13 (16.7 %)	9 (11 %)	14 (17.9 %)	12 (14.6 %)	30 (38.5 %)	33 (40.2 %)	18 (23.1 %)	23 (28 %)	3.60	3.73
7. “Our Health District staff, working with the County Health Department staff, considers continuous QI part of the public health agency’s working process.”	Intervention	5 (8.3 %)	5 (8.1 %)	8 (13.3 %)	3 (4.8 %)	16 (26.7 %)	4 (6.5 %)	16 (26.7 %)	27 (43.5 %)	15 (25 %)	23 (37.1 %)	3.47	3.97
	Control	3 (3.8 %)	2 (2.4 %)	9 (11.4 %)	10 (11.9 %)	14 (17.7 %)	10 (11.9 %)	32 (40.5 %)	33 (39.3 %)	21 (26.6 %)	29 (34.5 %)	3.75	3.92
8. “Our Health District staff, working with the County Health Department staff, continues to aim for change”	Intervention	6 (10.3 %)	5 (8.2 %)	4 (6.9 %)	2 (3.3 %)	15 (24.2 %)	6 (9.8 %)	22 (37.9 %)	26 (42.6 %)	11 (19 %)	22 (36.1 %)	3.48	3.95
	Control	4 (5 %)	4 (4.8 %)	5 (6.3 %)	8 (6.9 %)	15 (18.8 %)	9 (10.7 %)	36 (45 %)	34 (40.5 %)	20 (25 %)	29 (34.5 %)	3.79	3.90
9. “Our Health District staff work with the County Health Department staff to track progress continuously”	Intervention	6 (10.2 %)	6 (9.7 %)	6 (10.2 %)	5 (8.1 %)	19 (32.2 %)	8 (12.9 %)	19 (32.2 %)	28 (45.2 %)	9 (14.5 %)	15 (24.2 %)	3.32	3.66
	Control	5 (6.2 %)	5 (6.1 %)	6 (7.4 %)	5 (6.1 %)	15 (18.5 %)	13 (15.9 %)	34 (42 %)	36 (43.9 %)	21 (25.9 %)	23 (28 %)	3.74	3.82
10. “Information, ideas, and suggestions are actively exchanged at quality improvement meetings”	Intervention	6 (10 %)	5 (8.1 %)	8 (13.3 %)	5 (8.1 %)	12 (20 %)	5 (8.1 %)	20 (33.3 %)	22 (35.5 %)	14 (23.3 %)	25 (40.3 %)	3.47	3.92
	Control	7 (8.5 %)	8 (9.4 %)	10 (12.2 %)	7 (8.2 %)	26 (31.7 %)	22 (25.9 %)	29 (35.4 %)	34 (40 %)	10 (12.2 %)	14 (16.5 %)	3.30	3.46

Increases in support for prioritizing success for public health essential services quality improvement were greater among the PBRN-QI districts. The odds were 2.68 to 1 (*p* = .095) that PBRN-QI districts increased (40 % increase) in level of agreement with prioritization of essential services quality improvement (Item 2), compared to the Non-PBRN districts (6 % increase). Increased perception that Health District staff were motivated to implement changes for quality improvement (Item 3) were reported by PBRN-QI districts (49 % increase). This increase had a 4.96 odds ratio (*p* = .006) compared to the Non-PBRN districts (10 % increase).

### Use of data to inform decision making

Increases in QI organizational characteristics related to use of data to inform decision making were also statistically significant in comparing the PBRN- QI districts versus the Non-PBRN districts. The odds of increased agreement for “quality improvement goals are readily measurable” were 3.82 times greater (*p* = .024) for the PBRN-QI districts (42 % increase) than the Non-PBRN districts (3 % increase). Similarly, the PBRN-QI districts had 2.96 times greater odds (*p* = 0.058) of increased agreement (47 % increase) related to use of measurement to plan change (Item 6), compared to increases (13 %) in the non PBRN QI districts.

Ongoing (continuous) process: PBRN-QI districts also were statistically more likely to report increases in ***continuous*** QI processes. PBRN-QI supported districts had one year reported increases (53 % increase) in continuous QI being part of the public health agency's working process (Item 7), compared to the Non-PBRNQI districts (16 % increase), resulting in a 2.78 odds ratio (*p* = .006). Similarly the PBRN-QI districts were 4.71 times more likely (*p* = .011) to have increased (34 %) agreement that staff track progress continuously (Item 9), compared to the Non PBRN supported districts increase (4 %). The odds for PBRN-QI districts were slightly greater but non-significant (OR = 1.97) to have increased (48 %) agreement that district and CHD staff continued to aim for change (Item 8), compared to the Non-PBRN districts (13 % increase).

### Quality team collaboration

PBRN-QI districts also had increases in perceived agency-wide multidisciplinary collaboration. The odds of increase for this item (4) were 5.15 times greater (*p* = .07 for the PBRN-QI districts (58 % increase), compared to the Non-PBRN districts (no increase). The odds (OR = 2.12) for the PBRN-QI districts to report increases (48 %) compared to the Non-PBRN districts (15 % increase) for active exchange of information, ideas, and suggestions during quality improvement meetings (Item 10) were not significant at the .05 *p* value. See Table 4 for the responses to each item showing that the PBRN-QI responses frequently started at a lower level of assessed QI culture than the comparison group, but typically exceeded the comparison group after the one year study.

**Fig. 1:**
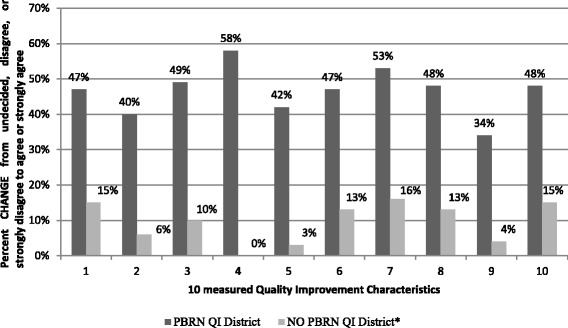
Comparison of change/increase in self-assessed organizational characteristics of QI culture of PBRN QI supported districts versus No PBRN QI supported districts during 12 months

Figure 1 illustrates the differences in QI culture change items between the PBRN supported QI districts and the comparison districts. In summary, health districts that participated in the PBRN supported QI activities reported a greater improvement in all ten gauges of organizational QI characteristics (five of which were statistically significant at *p* = 0.05) than the health districts that were not part of the PBRN supported QI efforts after adjusting for clustering/nesting using the multi-level mixed effects logistic model.

## Discussion

A major goal of QI is to move beyond individual QI projects to a change in the organization’s culture that reflects ongoing and pervasive application of QI principles and practices throughout the organization. Riley and others refer to this cultural transformation as Big QI [45, 46]. Kilmann et al. [46] define culture as the “shared philosophy, ideologies, values, assumptions, beliefs, expectations, attitudes and norms that knit a community together”. More simply, organizational culture is “the way things are done around here”, ([47], p5) but culture (passing on of values beliefs and behaviors) is by its nature, resistant to change. Incidental or rare episodic organizational behaviors would not be considered culture unless those behaviors were systematically perpetuated as is implied with definitions of culture. Similarly, a QI project can be successful in achieving the intended improvements without necessarily impacting the organizational capacity beyond that project. In other words, a single QI project does not create a culture which by definition has to do with passing on beliefs, practices and behaviors, or impacting the way the organization performs in the future.

Overcoming barriers to providing quality essential services can involve challenges in the way organizations do business. The top down, bureaucratic nature of some government agencies, described as barriers to QI in the introduction, may be considered the culture of many public agencies. Changing that culture through the use of QI may be much more important than a single QI project’s outcomes. The items selected for the QI instrument used for this study reflect key QI principles for public health agencies and the antithesis of organizational barriers to QI organizational culture.

Previous research had confirmed the local public health leadership perceptions that public health districts in Georgia played a pivotal role in providing essential services and demonstrated the potential role of districts as QICs [19]. The agencies engaged in the PBRN supported QI appeared to accomplish an improved QI organizational culture even when in one instance the specific QI project outcomes were not achieved. Clearly, district-based QI efforts had an impact on agency staff, at least on staff that came in contact with the QI project. While multiple projects involving staff across the district may be necessary to fully accomplish a QI culture transformation, clear evidence of change was documented with these efforts.

A limitation of the study was the lack of control over contamination by other QI efforts. Survey results showed QI culture improved across all Districts. This should not be surprising since the emphasis on QI within PHAB accreditation, CDC initiatives, and the Georgia Department of Public Health support, were likely to influence agency use of QI throughout Georgia. A few districts had extensive QI programs independent of the PBRN based initiative and the National Network of Public Health Institutes also sponsored QI initiatives in Georgia. Despite potential for all districts to receive encouragement to conduct QI, major percentage differences in changes of QI culture between PBRN-QI and Non-PBRN districts were dramatic and still statistically significant for five of the ten items after adjusting for clustering associated with District membership.

Another limitation was the effects of nesting or clustering due to multiple counties and survey respondents within each District. In effect, each survey response is not independent, which is a basic assumption of many statistical analyses. Clustering can also be a common concern with PBRN studies [48]. However, computerized programming has facilitated robust statistical analyses that adjust for the clustering effect by computationally adjusting the sampling error for lower variability within clusters compared to the overall variability, similar to how software adjusts for school and classroom clusters used within the Youth Risk Behavior Surveillance (YRBS) sampling and analysis. We used multi-level mixed effects logistic model to adjust for the effect of clustering due to District membership in computing statistical significance.

Another limitation of the study was the relatively small number of counties and districts involved with the study. In effect, the study was underpowered to detect smaller differences, especially after adjusting for clustering of CHDs within districts. The large differences between the PBRN-QI and the Non-PBRN responses for five of the items were statistically significant despite being underpowered to detect differences. Differences between the PBRN-QI and Non-PBRN responses for the other five items were not statistically significant. However these differences were substantial, and the insignificance may be due to the under-powering of the sample size after taking clustering into consideration.

Despite an emphasis on QI culture or “Big QI” by leaders of QI in public health [44, 45], very little effort to systematically study QI culture at the local level of public health has been reported. The Beitsch et al. [49] and Joly et al. [50–52] efforts to assess and monitor organizational QI culture in public health agencies are notable exceptions. However, their work with the evaluation of RWJF supported multi-state learning collaboratives is based on instruments developed to evaluate those multi-state efforts [52], and had exceptionally low construct validity (*r* = .18) for the cultural dimensions construct [52]. In contrast, development of the instrument used for this study was based on work to identify specific characteristics of QICs [18], and then modified for use with Georgia public health districts as public health QICs for local public health agencies [19].

Recent agency survey work using a QI culture instrument developed by Verma and Moran [53], based on literature review without reported validation, had overlapping constructs with the constructs reported here. “Elements” of culture that they examined included team work, employee empowerment, continuous process improvement and leadership commitment. Notably absent from this Verma-Moran instrument [53] or the studies by Joly [52] and colleagues were measures for “using data to inform decision making”, which some QI authorities consider to be a critical principle of QI [54] and an essential characteristic of a QI culture.

## Conclusions

QI continues to be supported and reinforced by accreditation efforts [55, 56]. Although QI is an underlying foundation for public health agency accreditation, accreditation processes do tend to focus on documentation of specific use of QI methods, However, this is one of several recently emerging attempts to assess changes in QI culture within local public health agencies. If a QI culture, or Big QI, is a major goal of public health quality improvement, it would appear that the basic principles of QI would demand some level of performance measurement and monitoring to assess the improvements in priority QI organizational cultural characteristics. Measuring QI culture that reflects basic principles of QI has a different focus than simply counting the number of QI methods such as PDSA cycles, control charts or root cause analysis.

This study also showed a positive impact of PBRN supported QI interventions on the QI organizational culture of local public health agencies in Georgia. In addition to the advances in studying QI organizational culture, this study provides important evidence for the value of PBRNs [56] in addressing major challenges to improving local public health systems [1–5] through highly applied research in practice settings.
